# The fasciola cinereum of the hippocampal tail as an interventional target in epilepsy

**DOI:** 10.1038/s41591-024-02924-9

**Published:** 2024-04-17

**Authors:** Ryan M. Jamiolkowski, Quynh-Anh Nguyen, Jordan S. Farrell, Ryan J. McGinn, David A. Hartmann, Jeff J. Nirschl, Mateo I. Sanchez, Vivek P. Buch, Ivan Soltesz

**Affiliations:** 1 Department of Neurosurgery, Stanford University, Stanford, CA USA; 2 Department of Pharmacology and the Vanderbilt Brain Institute, Vanderbilt University, Nashville, TN USA; 3 F.M. Kirby Neurobiology Center and Rosamund Stone Zander Translational Neuroscience Center, Boston Children’s Hospital, Harvard Medical School, Boston, MA USA; 4 Department of Neurology and Neurological Sciences, Stanford University, Stanford, CA USA; 5 Department of Pathology, Stanford University, Stanford, CA USA; 6 Yusuf Hamied Department of Chemistry, University of Cambridge, Cambridge, UK

**Keywords:** Epilepsy, Epilepsy

## Abstract

Targeted tissue ablation involving the anterior hippocampus is the standard of care for patients with drug-resistant mesial temporal lobe epilepsy. However, a substantial proportion continues to suffer from seizures even after surgery. We identified the fasciola cinereum (FC) neurons of the posterior hippocampal tail as an important seizure node in both mice and humans with epilepsy. Genetically defined FC neurons were highly active during spontaneous seizures in epileptic mice, and closed-loop optogenetic inhibition of these neurons potently reduced seizure duration. Furthermore, we specifically targeted and found the prominent involvement of FC during seizures in a cohort of six patients with epilepsy. In particular, targeted lesioning of the FC in a patient reduced the seizure burden present after ablation of anterior mesial temporal structures. Thus, the FC may be a promising interventional target in epilepsy.

## Abstract (web summary)

The posterior hippocampal tail is an important seizure node in humans and may be a promising interventional target in epilepsy.

## Main

Epilepsy is one of the most prevalent neurological disorders in the world, with tens of millions of people burdened by the occurrence of chronic spontaneous seizures^1^. For the one-third of patients with epilepsy who do not experience adequate seizure control with existing anti-seizure medications, surgical resection or ablation of the underlying epileptic tissue is the standard of care. Mesial temporal lobe epilepsy (TLE) is a common form of drug-resistant epilepsy, and the most common type treated with surgery, which ablates the anterior hippocampus and amygdala^2–8^. However, about one-third of patients who undergo surgery still do not receive adequate seizure freedom^2,9–13^. This raises the question of whether a seizure focus remains in the posterior hippocampus.

## Results

### The fasciola cinereum is highly active during seizures in mice

**Fig. 1:**
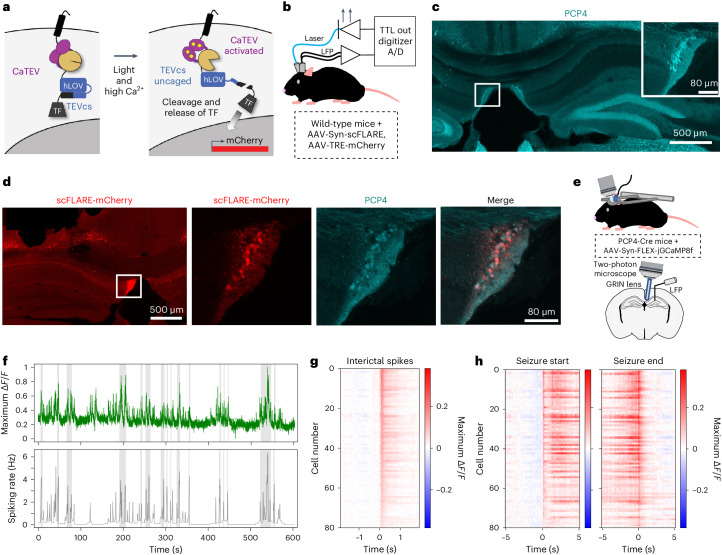
FC neurons are highly active during seizures in mouse models of acute and chronic TLE. **a**, Diagram showing how scFLARE induces stable labeling of neurons with mCherry only when there are both high intracellular Ca^2+^ and light delivery. CaTEV, Ca^2+^-activated TEV protease; hLOV, hybrid light-oxygen-voltage-sensing domain; TEVcs, Tobacco Etch Virus protease cleavage site; TF, transcription factor. **b**, Schematic showing closed-loop seizure detection and light delivery in wild-type mice to label seizure-active neurons. TTL, transistor–transistor logic **c**, Coronal section wide-field image and higher magnification of the boxed region of interest (inset) from a non-epileptic mouse, with evident PCP4 expression in the FC. Representative image from three mice, three to five sections per mouse. **d**, Coronal section wide-field image (left) and higher magnification of the region of interest (right three images) with FC cells labeled with mCherry, indicating that the FC cell population is highly active during seizures. Representative image from six mice, three to five sections per mouse. The scale bar in the fourth image also applies to the second and third images. **e**, Schematic showing two-photon imaging setup in PCP4-Cre chronically epileptic mice. **f**, Example trace showing the fluorescence intensity (Δ*F*/*F*, normalized to that cell’s maximum) of a representative FC neuron imaged using jGCaMP8f (green), above a plot of spiking rate recorded simultaneously from that mouse’s hippocampus, with time intervals meeting seizure criteria highlighted in gray. **g**, Heat map for each of the 80 FC neurons recorded from three mice, showing an increase in activity (as reflected by intracellular Ca^2+^ and Δ*F*/*F*) during interictal spikes, set as time = 0. **h**, Heat map for each of the recorded FC neurons showing an increase in activity during the start of a seizure and a decrease in activity when the seizure ends. Correlation between Ca^2+^ activity and spiking for the cells is *r* = 0.43 ± 0.03 (s.e.m.).

**Extended Data Fig. 1:**
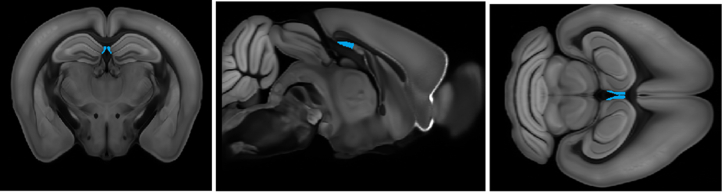
FC location in mouse brain. Coronal, sagittal, and axial images of mouse FC (blue) based on Allen Mouse Common Coordinate Framework^27^.

**Extended Data Fig. 2:**
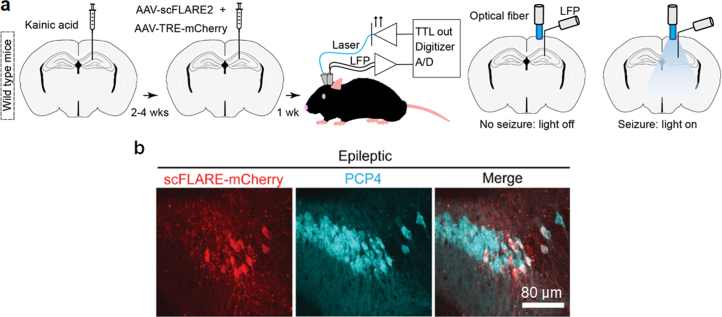
FC neurons are highly active during seizure in a mouse model of chronic temporal lobe epilepsy. **a**, Schematic showing the injection of kainic acid into the hippocampus of wild-type mice to generate chronic epilepsy, followed by viral injection 2–4 wks later to induce expression of the scFLARE tool and mCherry reporter in the hippocampus. Mice then underwent insertion of a hippocampal wire electrode to detect seizures and an optical fiber. Light was delivered through the optical fiber when the electrode detected a seizure. **b**, In epileptic mice, scFLARE-mCherry labeled PCP4+ FC neurons. Representative image from 3 mice, 3–5 sections per mouse. Of note, there is not significant cell death of the PCP4+ neuron population within the FC in this model.

**Extended Data Fig. 3:**
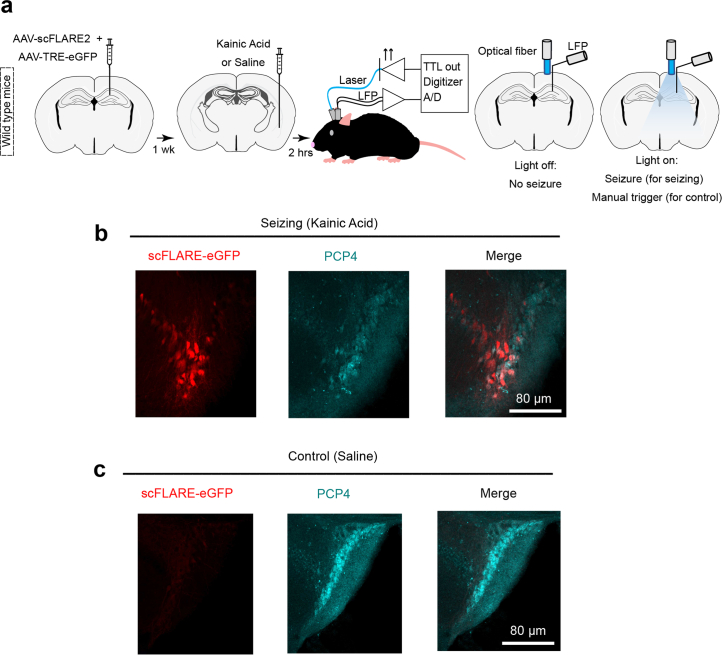
FC neurons are highly active during seizure in the intra-amygdalar kainic acid mouse model of temporal lobe epilepsy. **a**, Schematic showing the injection of virus to induce expression of the scFLARE tool and eGFP reporter in the hippocampus of wild-type mice, followed 1 week later by injection of kainic acid into the amygdala, along with insertion of a hippocampal wire electrode to detect seizures and an optical fiber. Light was delivered through the optical fiber when the electrode detected a seizure. **b**, In seizing mice, scFLARE-eGFP labeled PCP4+ FC neurons. Representative image from 3 mice, 3 sections per mouse. **c**, In non-seizing mice (injected with saline rather than kainic acid), scFLARE-eGFP had neglible labeling of PCP4+ FC neurons. Representative image from 2 mice, 3 sections per mouse.

We have recently shown that a light- and calcium-gated molecular integrator called scFLARE (single-chain fast light- and activity-regulated expression; Fig. 1a) can be used to label neurons active during seizures in mice^14^. To identify novel brain regions involved in TLE, we first transduced the hippocampus of mice with scFLARE and mCherry-reporter viruses before intrahippocampal injection of kainic acid to induce acute seizures. We then used a closed-loop system to deliver a pulsed bout of blue light in response to a detected seizure on the hippocampal local field potential (LFP) recordings to activate the scFLARE tool and enable the labeling of seizure-active neurons (Fig. 1b). Of the mCherry-positive areas in the hippocampus^14^, we found prominent scFLARE-mediated labeling of cells in the fasciola cinereum (FC) subregion (Fig. 1c,d), which forms a longitudinal midline structure in the dorsal hippocampus of rodents^15–17^ (Extended Data Fig. 1) and prominently expresses the genetic marker Purkinje cell protein 4 (PCP4; Fig. 1c). A recent study in non-epileptic rats found FC neurons to be distinct from adjacent brain areas in their morphology and in their connectivity^16^, including input from the lateral entorhinal cortex and output to the crest of the dentate gyrus. Importantly, the labeling of FC cells was also found in scFLARE experiments performed in chronically epileptic mice (Extended Data Fig. 2) and in mice with acute seizures due to intra-amygdalar kainic acid injection (Extended Data Fig. 3a,b). Negligible scFLARE-mediated labeling was seen in the FC of non-seizing control mice (Extended Data Fig. 3c).

**Extended Data Fig. 4:**
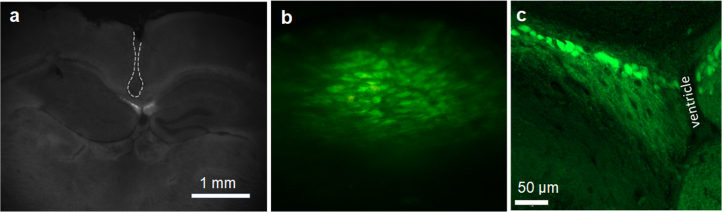
Calcium sensor expression in FC. **a**, Coronal section fluorescence image of Cre-dependent jGCaMP8f expressed in the FC of PCP4-Cre mice, with outlined tract of GRIN lens aiming at FC of hippocampus ipsilateral to kainic acid injection. **b**, 2-photon microscope (average intensity projection) image through GRIN lens of individual cells. Representative image from 3 mice. **c**, Post-hoc image (coronal section) shows the characteristic granule cell-like morphology and elongated processes of FC neurons (same mouse as panels a and b). Representative image from 3 mice, 3–5 sections per mouse.

To study the patterns of FC neuronal dynamics during epileptic activity, we performed two-photon calcium imaging of FC neurons by using a PCP4-Cre transgenic mouse line, where Cre recombinase is selectively expressed in PCP4 expressing neurons, and Cre-dependent expression of the genetically encoded calcium indicator jGCaMP8f in the FC of chronically epileptic mice. These mice were also implanted with an LFP electrode placed in the hippocampus to record interictal and ictal activity (Fig. 1e,f). We confirmed accurate targeting of FC neurons by their distinct granule-cell-like morphology and dendritic branches fanning out toward the ventricle^16^ (Extended Data Fig. 4). We found increases in calcium activity of FC neurons aligned with the epileptiform spiking activity (Fig. 1g,h). Analysis of individual cell calcium dynamics showed that FC neuron activity increased during interictal spikes (Fig. 1g) and at the start of seizures (Fig. 1h, left panel), and decreased when the seizure ended (Fig. 1h, right panel). Together, these results show that FC neurons are highly active members of the TLE seizure network.

### The FC is a therapeutic target in mice with epilepsy

**Fig. 2:**
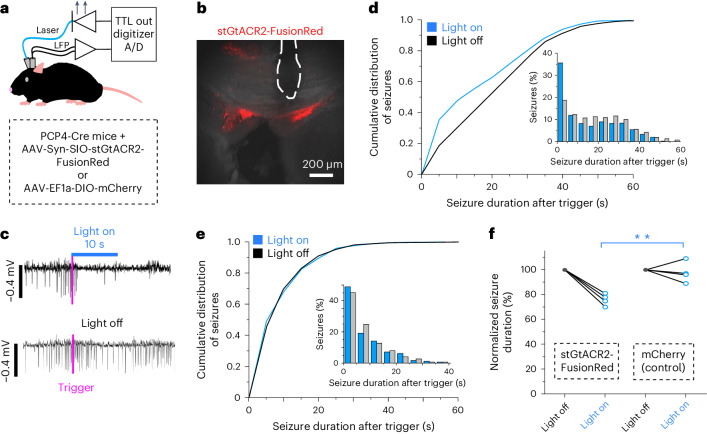
The FC in mice is an intervenable target for the treatment of TLE. **a**, Schematic showing closed-loop seizure detection and light delivery to activate the inhibitory opsin stGtACR2 in PCP4-Cre mice. **b**, Section of mouse hippocampus showing stGtACR2–FusionRed in the bilateral FC of a chronically epileptic mouse, with the tract (dotted white line) left by the optical fiber terminating just superior to the FC ipsilateral to the previous kainic acid injection. Representative image from four mice, three to five sections per mouse. **c**, Example seizures detected at the time marked by pink lines in which light was delivered (top, blue bar) and not delivered (bottom). **d**, Cumulative distribution curve and histogram (*n* = 4 mice) showing a greater proportion of short seizures (<5 s) measured from the time of seizure detection when light is delivered (blue) compared with when it is not (gray) for PCP4-Cre mice expressing stGtACR2 in the FC. A mixed-effect model comparing the seizure durations with light off versus light on resulted in *F*(1, 1,010) = 51.47, *P* < 0.0001, for mice expressing stGtACR2. **e**, Cumulative distribution curve and histogram (*n* = 4 mice) showing similar seizure duration after the trigger when light was delivered (blue) compared with when it was not (gray) for PCP4-Cre control mice expressing mCherry in the FC. A mixed-effect model comparing the seizure durations with light off versus light on resulted in *F*(1, 778) = 0.1133, *P* = 0.74, for control mice expressing mCherry. **f**, Normalized seizure duration comparing seizure length with light off versus that with light on, for mice expressing stGtACR2 and control mice expressing mCherry in the FC. stGtACR2, 76 ± 3% (2,075 seizures from 4 mice); mCherry, 98 ± 4% (1,627 seizures from 4 mice); normalized seizure duration ± s.e.m. **P = 0.0038, *t* = −4.56213, two-tailed *t*-test.

**Extended Data Fig. 5:**
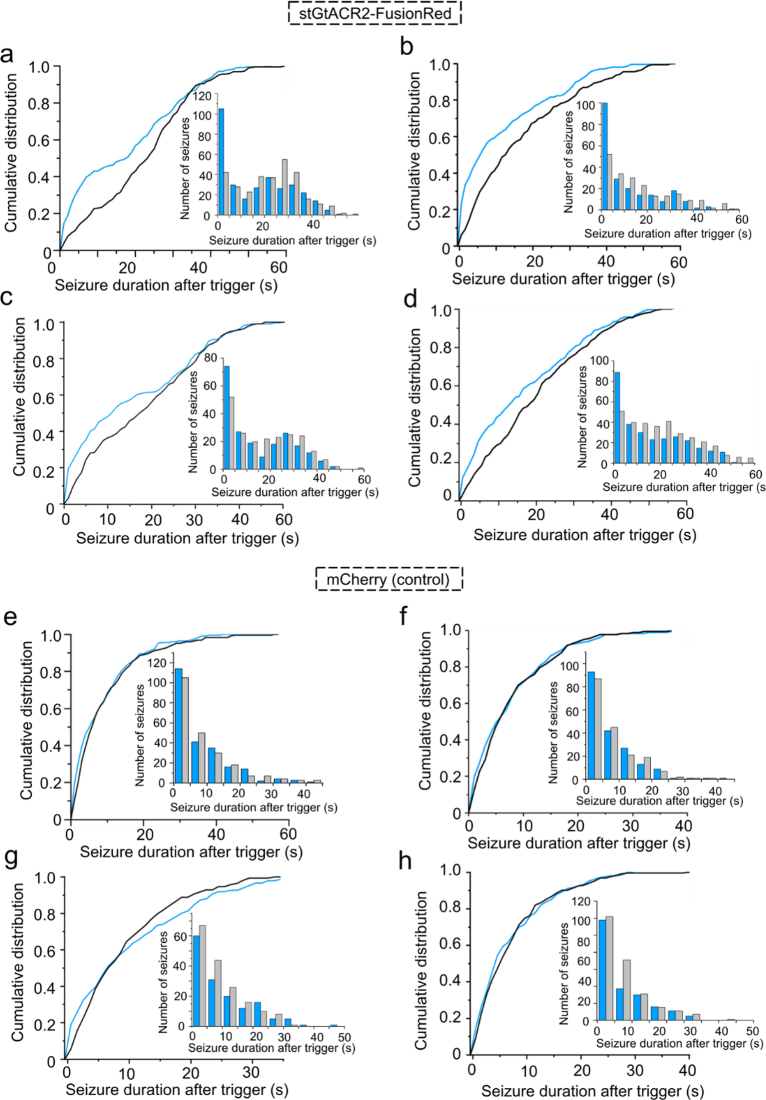
Effect of optogenetic inhibition of FC neurons on seizure duration for individual mice. **a**–**d**, Cumulative distribution curves and histograms for each of four individual mice demonstrating a greater proportion of short seizures (<5 sec) measured from the time of seizure detection when light is delivered (blue) compared to when it is not (gray) for PCP4-Cre mice expressing stGtACR2 in the FC. Numbers of seizures detected for each animal, listed as # of seizures with light on / # of seizures with light off, are as follows: a. stGtACR2 mouse 1: 313/304. Mann-Whitney test comparing distributions: P < 0.00001. b. stGtACR2 mouse 2: 208/216. Mann-Whitney test comparing distributions: P < 0.00001. c. stGtACR2 mouse 3: 210/215. Mann-Whitney test comparing distributions: P < 0.01. d. stGtACR2 mouse 4: 291/318. Mann-Whitney test comparing distributions: P < 0.0001. **e**–**h**, Cumulative distribution curves and histograms for each of four individual mice demonstrating similar seizure duration after trigger when light is delivered (blue) compared to when it is not (gray) for PCP4-Cre control mice expressing mCherry in the FC. Numbers of seizures detected for each animal, listed as # of seizures with light on / # of seizures with light off, are as follows: e. Control mouse 1: 230/238. Mann-Whitney test comparing distributions: P = 0.12. f. Control mouse 2: 188/183. Mann-Whitney test comparing distributions: P = 0.39. g. Control mouse 3: 151/172. Mann-Whitney test comparing distributions: P = 0.65. h. Control mouse 4: 217/258. Mann-Whitney test comparing distributions: P = 0.40.

To test whether FC neurons have a mechanistic role in seizure propagation (and would thus be a potential target for intervention), we used an optogenetic approach and expressed the inhibitory opsin soma-targeted *Guillardia* theta anion-conducting channelrhodopsin 2 (stGtACR2) in FC neurons of chronically epileptic PCP4-Cre mice. An optical fiber was inserted to terminate superior to the FC, while an LFP electrode was placed in the hippocampus to enable closed-loop seizure detection to trigger optogenetic inhibition (Fig. 2a–c). Closed-loop light delivery shortened seizure duration in chronically epileptic mice expressing stGtACR2 in FC neurons (Fig. 2c,d,f), but not in those without the opsin (Fig. 2e,f). This effect is evident not only in the aggregated data but also in the individual responses of each of the four mice in the GtACR2 and control groups (Extended Data Fig. 5). Thus, FC neurons are highly active during seizures, and their inhibition can provide seizure control in mice.

### The FC is a node for seizure propagation in humans

**Fig. 3:**
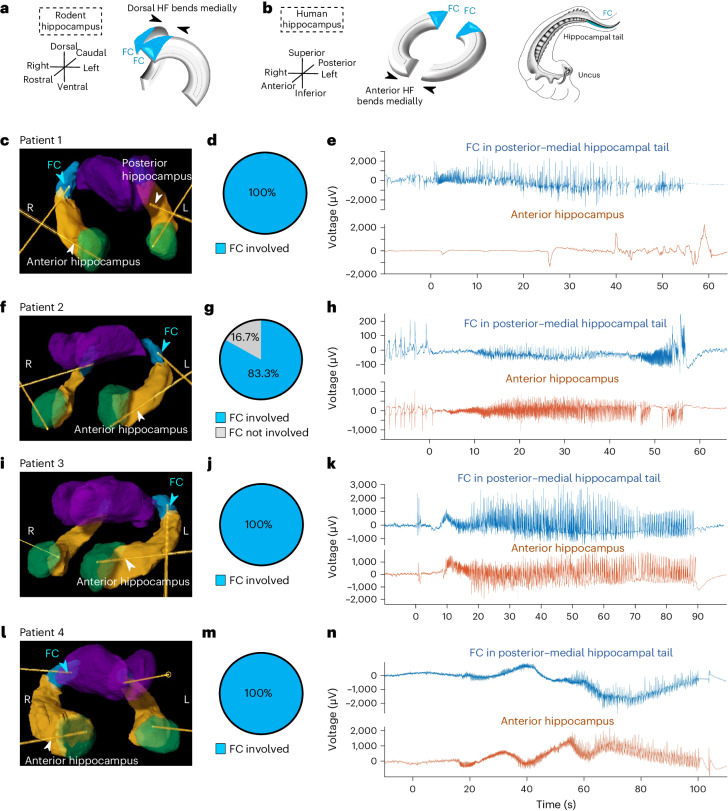
The FC in the human posterior–medial hippocampal tail is involved in seizure initiation and propagation in TLE. **a**,**b**, Schematic illustrations of hippocampal formation (HF) comparing rodent and primate FC anatomy, as previously described^20^. **a**, The rodent dorsal HF bends medially, leading to a medially located FC (blue). **b**, In primates, the anterior (equivalent to ventral) HF (uncus and genu of the anterior hippocampus) bends medially, so the FC is posteriorly located. Hippocampus drawing adapted with permission from ref. ^18^, Springer. **c**, Diagram of sEEG electrodes in the right posterior–medial hippocampal tail (FC, blue), the hippocampal body and head (orange), and the amygdala (green) of patient 1, with the thalamus shown in purple. Of note, the left hippocampus of this patient has an electrode targeting the hippocampal tail less posteriorly than the FC, in a more conventional trajectory. **d**, All seizures recorded from this patient had FC involvement (*n* = 7 of 7 seizures). **e**, LFP trace for a representative seizure as recorded by electrodes in the FC (blue) and the amygdala and anterior hippocampus (orange). **f**, Diagram of sEEG electrodes targeting the amygdala, anterior hippocampus and FC of patient 2. **g**, Of the seizures recorded from this patient, 83% had FC involvement (*n* = 15 out of 18 seizures). **h**, Representative LFP traces for a seizure from this patient. **i**, Diagram of sEEG electrodes targeting the amygdala, anterior hippocampus and FC of patient 3. **j**, All seizures recorded from this patient had FC involvement (*n* = 5 out of 5 seizures). **k**, Representative LFP traces for a seizure from this patient. **l**, Diagram of sEEG electrodes targeting the amygdala, anterior hippocampus and FC of patient 4. **m**, All seizures recorded from this patient had FC involvement (*n* = 3 out of 3 seizures). **n**, Representative LFP traces for a seizure from this patient. Note the differences in the vertical axes in **e**, **h**, **k** and **n**.

We then sought to determine whether FC plays a similar role in human patients with TLE. In humans, the FC (along with the fasciolar gyrus) is the main gray matter structure in the posterior–medial hippocampal tail at the level of the quadrigeminal cistern, posterior to the brain stem and inferior to the corpus callosum^18,19^. The mouse hippocampus bends medially in its dorsal aspect, resulting in the medial position of the mouse FC^20^ (Fig. 3a). The comparable medial bend in the human hippocampus occurs ventrally instead (that is, anteriorly, as the human hippocampus also lies in a more horizontal rather than vertical orientation) at the level of the uncus^20^ (Fig. 3b). The dorsal position of the FC thus results in it being in the posterior hippocampal tail^18^ (which also has a gentle medial bend; Fig. 3b), rather than being along the midline as in mice.

**Fig. 4:**
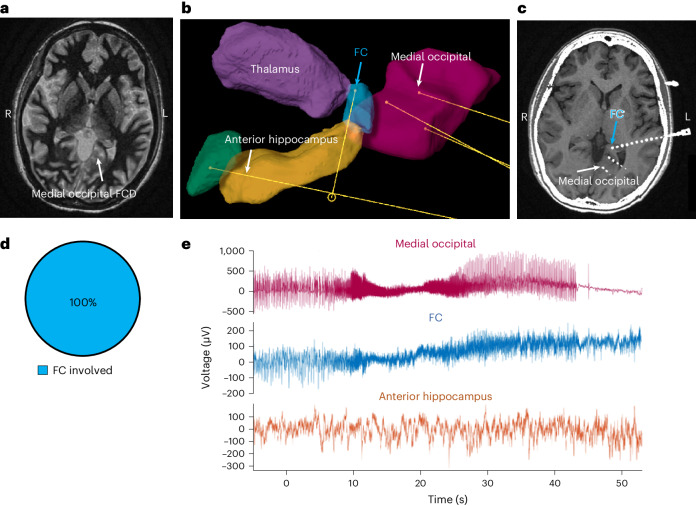
FC is involved in seizures originating from an occipital focus. **a**, Fast gray matter acquisition T1 inversion recovery axial MRI showing left occipital FCD. **b**, Diagram of sEEG electrodes in the left posterior–medial hippocampal tail (FC, blue), as well as in the hippocampal body and head (orange) and amygdala (green), of patient 5, with the thalamus shown in purple. Other electrodes targeted the medial–occipital FCD in the left lingual gyrus (pink). **c**, Reconstruction of sEEG electrode positions based on postoperative CT and T1 MRI, with the blue arrow pointing to the contact in the FC. The electrode in the occipital FCD is also apparent, indicated by the white arrow. **d**, All seizures recorded from this patient had FC involvement (*n* = 15 out of 15 seizures). **e**, LFP trace for a representative seizure originating from the medial–occipital FCD, as recorded by electrodes in the medial–occipital cortex and FCD (pink), FC (blue) and anterior hippocampus (orange).

**Fig. 5:**
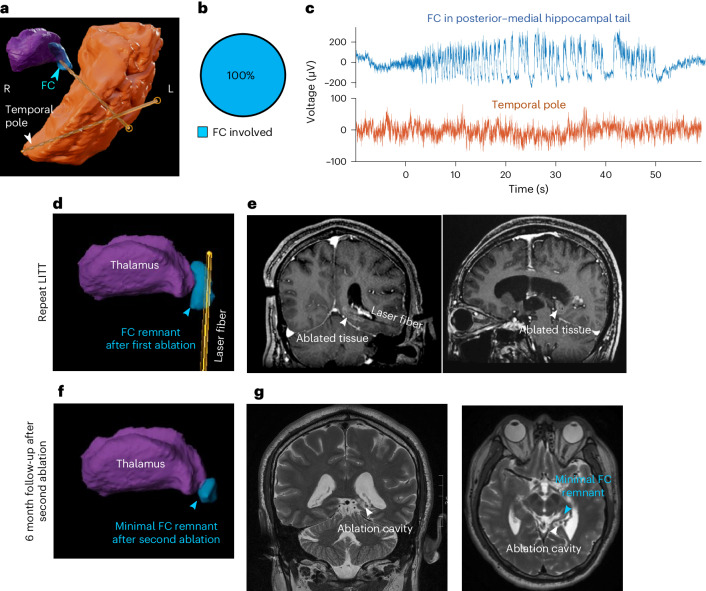
The FC is a viable treatment target in humans. **a**, Reconstruction of sEEG electrodes (yellow) in remnants of the hippocampal tail (blue), as well as in the temporal pole (orange) of patient 6 who had a previous amygdalohippocampectomy. The thalamus is shown in purple. **b**, All seizures recorded from this patient had FC involvement (*n* = 117 out of 117 seizures). **c**, LFP trace for a representative seizure originating from the posterior–medial hippocampal tail remnant (onset time at 0 s), as recorded by electrodes in the FC (blue) and in the temporal pole (orange). **d**,**e**, Three-dimensional reconstruction (**d**) and intraoperative in-line and orthogonal slice of post-contrast T1 MRI (**e**) showing a laser fiber inserted in the lateral edge of the hippocampal tail to avoid heat spread into the thalamus. The contrast-enhancing lesion appears to include the entirety of the remnant. **f**,**g**, Three-dimensional reconstruction (**f**) based on coronal and axial T2-weighted MRI (**g**) at 6 months following the second ablation, with a small residual FC adjacent to the lateral geniculate nucleus of the thalamus.

Before this study, there has not been sufficient suspicion of the FC as a specific seizure focus^13^ to systematically target it during stereoelectroencephalography (sEEG) in human patients. Here we present six patients (Table 1, and Figs. 3c–n, 4 and 5) who underwent sEEG implantation where posterior hippocampus sampling was desired, specifically targeted to the FC. In humans, sEEG sampling of the hippocampus rarely extends that far posterior–medially along the tail; the posterior hippocampus is usually targeted where it is larger and thicker, anterior to the FC. This trade-off is explicit for patient 1 (Fig. 3c), whose anatomy allowed only the FC to be targeted on the right, while a more conventional trajectory to the hippocampal tail was used on the left. For each patient, sEEG widely sampled many brain regions (such as frontal, temporal, insular, occipital and thalamic) that were suspected to be involved in the seizure network of each patient based on a consensus of imaging, non-invasive EEG, semiology and neuropsychiatric testing reviewed by a panel of clinicians.

**Table 1:** Patient characteristics and sEEG findings for patients with electrodes planted in the FC, other mesial temporal structures and other non-temporal structures

Patient	1	2	3	4	5	6
**Age (years)**	28	38	47	36	48	51
**Sex**	Male	Male	Female	Male	Female	Male
**Seizure frequency before treatment**	4–5 per week	1 per week	3 per week	1–5 per year	2 per month	3–4 per month (before the first ablation); 2 per month (after the first ablation, before the second)
**Semiology**	Impaired awareness with lip smacking and bilateral hand writhing	Fingertip tingling, lip smacking, behavioral arrest	Reflex seizure triggered by eating and brushing of teeth: lip smacking, stereotyped R hand movements, impaired consciousness	Loss of consciousness with head turning and gaze deviation to the left, bilateral tonic–clonic activity, tongue biting, incontinence	Loss of awareness, staring, lip smacking, manual automatisms; has chronic R superior quadrantanopsia	Behavior arrest and convulsion, often out of sleep and occasionally preceded by right arm and leg stiffening
**Neuropsychiatric findings**	Low-average cognitive ability, overall non-lateralizing versus bilateral	Impaired auditory comprehension, short-term memory, attention, spelling; localizes L > R mesial temporal regions and frontotemporal and anterior temporal networks	High baseline with mildly impaired semantic verbal fluency (localizes to the L inferior frontal gyrus) but no memory impairment	Impaired verbal memory measures	High-average intelligence with average revealed abilities, high visuospatial skills, mild compromise of the L frontotemporal function	High visual spatial abilities and verbal skills, with overall cognitive and motor deficits localizing to the L frontotemporal focus
**Current ASMs**	Lamotrigine, lacosamide	Lacosamide, valproic acid, zonisamide	Lamotrigine	Lacosamide	Eslicarbazepine, levetiracetam	Cenobamate, levetiracetam
**Previous ASMs**	Levetiracetam, brivaracetam, phenytoin	Levetiracetam, oxcarbazepine, phenytoin	Topiramate, zonisamide	Carbamazepine, lamotrigine, levetiracetam, topiramate, valproic acid, zonisamide	Lamotrigine, oxcarbazepine	Lacosamide, lamotrigine, zonisamide
**MRI findings**	Normal (no sclerosis)	Bilateral MTS	Mildly increased T2/FLAIR intensity of the L hippocampus and throughout the L temporal lobe (possible sclerosis)	Normal (no sclerosis)	Medial L occipital cortical dysplasia and L hippocampal signal abnormality (possible sclerosis)	L mesial temporal sclerosis s/p LITT, with the residual posterior hippocampal tail
**PET findings**	Subtle decreased uptake of the L mesial temporal lobe	Severe symmetric hypometabolism of the anterior temporal lobes	Mild hypometabolism in the L hippocampus and insula	Normal	Decreased uptake in the L occipital focal cortical dysplasia and L temporal lobe	Normal
**Phase I (non-invasive video EEG) findings**	Bitemporal independent temporal sharps (L > R), multiple focal seizures with R temporal onset	Focal seizures with R temporal, and L temporal and insular, onset	Focal impaired awareness seizures with L mid-temporal onset; L anterior–middle temporal sharps, independent broader-field L posterior discharges; rare poorly formed R temporal sharps	R temporal focal to bilateral tonic–clonic seizures occurring out of sleep	Focal seizures with L temporal onset; central versus L temporal diffuse theta slowing	(After the first ablation, before the second) Behavioral arrest and bilateral tonic–clonic seizures of L temporal onset; intermittent L temporal slowing and temporal breach rhythm
**Phase II (sEEG) findings**	2 seizures, R FC independently involved; 4 seizures, R AH and R FC involved simultaneously; 1 seizure, L AH to R AH and R FC	15 seizures rapidly spread to FC from AH, 13 L AH to L FC and 2 R AH to R FC; 3 seizures, AH involved without FC	3 seizures spread to FC from AH (2 on R, 1 on L); 2 seizures with simultaneous onset in L FC and AH	3 seizures, R neocortical STG onset with spread to R AH and R FC	15 seizures, rapid spread to L FC from L occipital FCD, without spread to L AH	117 seizures originating from L FC only
**Diagnosis**	Bilateral mesial temporal epilepsy	Bilateral mesial temporal epilepsy with bilateral MTS	Bilateral mesial temporal epilepsy	Unilateral R neocortical TLE	Unilateral L neocortical occipital lobe epilepsy	Unilateral mesial temporal epilepsy with MTS
**Intervention**	Bilateral mesial temporal RNS^a^	Bilateral mesial temporal RNS^a^	Bilateral mesial temporal RNS^a^	Electrocorticography-guided tailored temporal lobe neocortical resection	Resection of the L occipital focal cortical dysplasia with cavity flanked by RNS leads	LITT of residual L FC

^a^Note that bilateral hippocampal patients require RNS rather than lesioning (that is, surgery or laser ablation), because both hippocampi cannot be lesioned without memory impairment. Due to the RNS systems being currently limited to two leads, bilateral mesial temporal RNS in these patients used conventional hippocampal trajectories focusing on the anterior hippocampus. Therefore, for patients 1–3, our knowledge of FC involvement did not change the initial management, but if systems with more leads become available, the FC could also be targeted with additional dedicated trajectories. R, right; L, left; AH, amygdala and anterior hippocampus; MTS, mesial temporal sclerosis; FCD, focal cortical dysplasia; RNS, responsive neurostimulation; ASM, anti-seizure medication; T2-FLAIR, T2-weighted-Fluid-Attenuated Inversion Recovery; s/p, status post.

**Extended Data Fig. 6:**
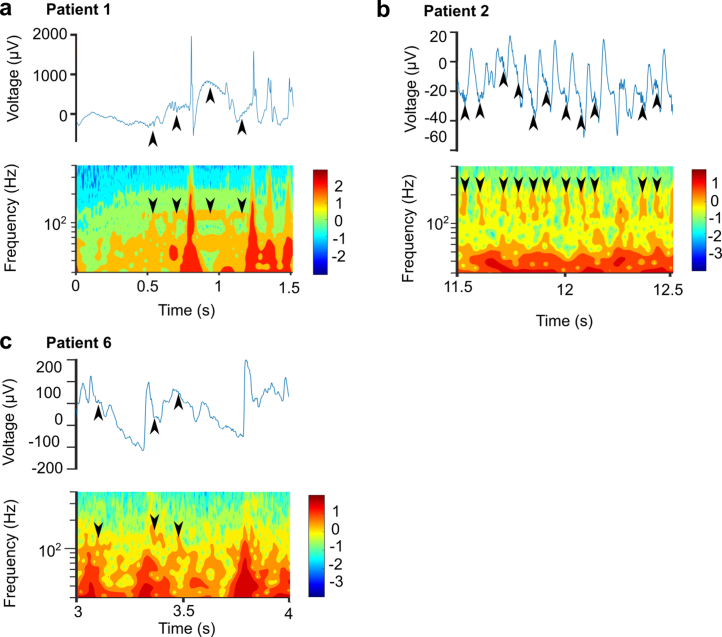
High frequency oscillations (HFOs) in FC. Excerpts of FC LFP tracings presented in Figs. 3 and 5, with shorter time scale (see x-axis). Below each LFP tracing are corresponding spectrograms, with black arrows indicating HFOs. **a**, LFP tracing and spectrogram from 0 s to 1.5 s for patient 1. **b**, LFP tracing and spectrogram from 11.5 s to 12.5 s for patient 2. **c**, LFP tracing and spectrogram from 3 s to 4 s for patient 6. Time-frequency plots were obtained via convolution with a Morlet wavelet function. An amplitude of the wavelet transform was then obtained via its absolute value, and displayed on a logarithmic scale (color map).

In sampled patients, the posterior–medial hippocampal tail containing the FC was a site of epileptiform discharges and ictal spiking, with high-frequency oscillations (80–250 Hz) consistent with participation in a seizure propagation network (Extended Data Fig. 6). The variety of stereotyped patterns for the involvement of the FC (independent involvement of only the FC or only the anterior hippocampus, simultaneous involvement of both or spread from the anterior hippocampus to the FC; Table 1) suggests that the FC is an important node for seizure propagation in mesial TLE. In addition, the FC may be important not only in mesial TLE but also in patients with temporal (patient 4) or non-temporal (patient 5) neocortical epilepsy. In both patients, seizures started in the neocortical tissue (patient 4—non-lesional superior temporal gyrus (STG); patient 5—occipital focal cortical dysplasia) and propagated to the FC. Particularly in patient 5, the FC activity was robustly independent of the anterior hippocampus (Fig. 4).

### The FC is a therapeutic target in humans with epilepsy

**Extended Data Fig. 7:**
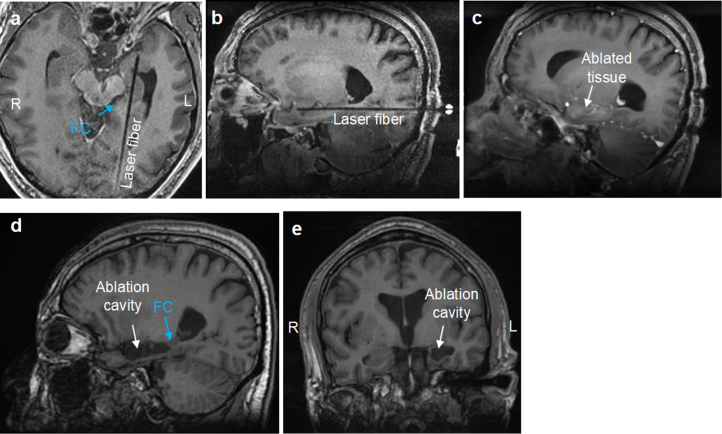
Prior LITT amygdalohippocampectomy for patient whose seizures recurred (Patient 6). **a**, **b**, Intraoperative in-line slices of T1 MRI demonstrating optical fiber inserted in along long axis of hippocampus and amygdala of patient 6. **c**, T1 post-contrast MRI immediately after ablation, demonstrating large rim-enhancing lesion that encompasses amygdala and hippocampal head and body. **d**, **e**, Follow-up T1 MRI demonstrating cavity where mesial temporal structures were successfully ablated, aside from the posterior-medial remnant of the hippocampal tail including the FC.

**Extended Data Fig. 8:**
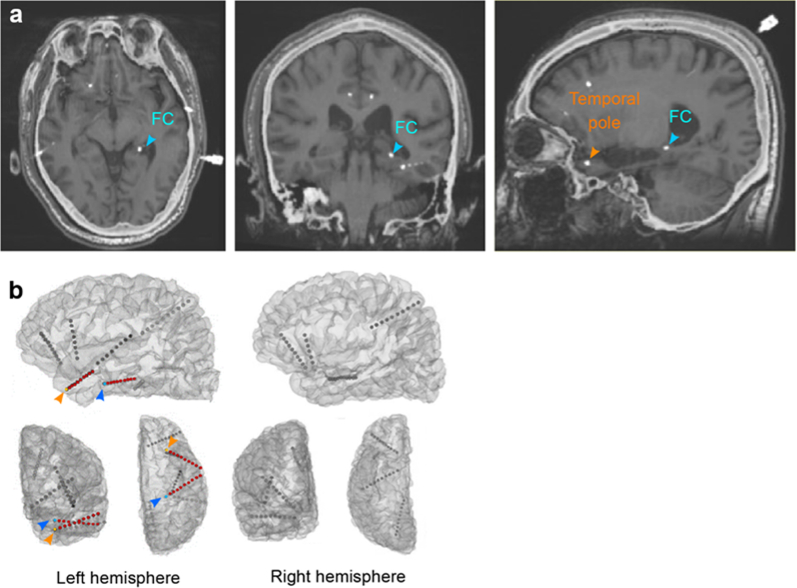
EEG sampling trajectories for Patient 6 after amygdalohippocampectomy. **a**, Reconstruction of sEEG electrode positions based on post-operative CT and T1 MRI, with blue arrow pointing to contact in FC. Electrode in temporal pole also visible in sagittal scan, noted by orange arrow. **b**, Diagram of sEEG electrode positions demonstrating wide sampling across both hemispheres, not restricted to mesial temporal structures. Contacts of the posterior hippocampal and temporal electrodes are red, with the FC contact highlighted in blue and the temporal pole electrode highlighted in orange. Blue arrow points to left FC, orange arrow points to left temporal pole.

In humans, open amygdalohippocampectomy resections rarely extend as far posterior–medially along the hippocampal tail to reach the FC. For laser interstitial thermal therapy (LITT) amygdalohippocampectomy, the human FC is medial of the posterior-to-anterior trajectory along the hippocampal long axis used to ablate the amygdala and the rest of the hippocampus. In a patient whose seizures recurred after LITT despite successful ablation of the amygdala and anterior hippocampus (Extended Data Fig. 7 and Table 1 (patient 6)), we hypothesized that the FC was responsible for his continued seizures. In a repeat evaluation including sEEG, we implanted one electrode in the FC, which was the expected remnant of the LITT procedure (Extended Data Fig. 8). Indeed, the sEEG localized his seizure onset zone exclusively to the FC (Fig. 5a–c). He then underwent a repeat laser ablation, which lesioned most (but not all) of the remaining FC (Fig. 5d–g). At the 18 month follow-up, the frequency of his seizures was reduced by 83% (from twice per month before FC ablation down to once every 3 months). Thus, the FC is a targetable source of seizure recurrence in humans.

## Discussion

Seizure freedom remains an elusive goal for a substantial proportion of patients with epilepsy. Precise seizure localization in patients often relies on sEEG, that is, electrodes implanted directly into the brain. Brain regions targeted by sEEG electrodes are often biased toward known epileptogenic foci, thus constraining the discovery and identification of novel seizure foci in humans. Here we used scFLARE in combination with closed-loop optogenetics to identify the FC subregion of the brain as a novel node of seizure propagation and target for intervention in mice and extended these insights to human patients. Critically, our finding that all patients with TLE from whom we obtained recordings had epileptic activity in this area can lead to a fundamental change in the standard of care. Approximately two-thirds of patients with mesial TLE who have a conventional lesioning (leaving the FC as a residual) have adequate seizure response; thus, it currently would be unethical to target only the FC without the anterior mesial temporal structures. However, conventional lesioning alone is inadequate for one in three patients. Our data currently support the FC as a target in patients with TLE for whom lesioning of the anterior hippocampus and amygdala was insufficient.

Our findings suggest that the FC should be targeted during sEEG for all patients with suspected TLE. In unilateral mesial TLE, FC seizure activity can inform the extent of lesioning required via surgical resection or laser ablation. For bitemporal epilepsy, patient and side-specific FC involvement could alter conventional approaches to the hippocampus for responsive neurostimulation, which currently miss the posterior hippocampal tail. Based on these results, FC involvement could inform surgical management to optimize outcomes for patients with medically refractory TLE.

## Methods

### Animals

All procedures were carried out in accordance with the National Institutes of Health guidelines for animal care and use and were approved by the Administrative Panel on Laboratory Animal Care of Stanford University (protocol number 30183). For the scFLARE experiments, adult wild-type male C57BL/6 mice 12–20 weeks old (Jackson Laboratory, strain number 000664) were used. For the two-photon microscopy and closed-loop optogenetic experiments, PCP4-Cre male and female mice 12–24 weeks old (RIKEN, strain number RBRC05662) were used.

All surgeries were conducted under aseptic conditions using a small-animal stereotaxic instrument (Leica Biosystems). Mice were anesthetized with isoflurane (5% for induction, 1.5–2.0% after) in the stereotaxic frame for the entire surgery, and their body temperature was maintained using a heating pad.

### Kainate injection in mice

Intrahippocampal kainic acid injections were performed as described previously^21^. Briefly, mice were placed under isoflurane anesthesia and given local anesthetic, 0.5% bupivacaine, at the site of incision. Kainic acid (60 nl, 20 mmol l^−1^ in saline; Tocris Bioscience) was injected into the dorsal hippocampus (from the bregma: −2.0 mm anterior-posterior (AP), +1.25 mm medial-lateral (ML), −1.6 mm dorsal–ventral (DV)). The above protocol was modified for intra-amygdala kainic acid injections in which 100 nl, 20 mmol l^−1^ kainic acid in saline was injected into the right basolateral amygdala (from the bregma: −1.2 mm AP, +3.3 mm ML, −4.2 from dura DV). For both intrahippocampal kainic acid and intra-amygdala kainic acid, kainic-acid-induced status epilepticus after injection was allowed to self-terminate. For experiments conducted in the setting of acute seizures, animals were allowed to recover for 2 h (scFLARE experiments in Fig. 1c,d and Extended Data Fig. 3b) or returned to the vivarium for at least 2 weeks to allow for the emergence of chronic spontaneous seizures (scFLARE experiments in Extended Data Fig. 2b, and all calcium imaging and closed-loop optogenetic experiments).

### Virus infusion in mice

For scFLARE experiments, the hippocampus was targeted using the following coordinates from the bregma: −2.3 mm AP, +1.5 mm ML and −1.35 mm DV. Adeno-associated viruses 1/2 (AAV1/2s) carrying scFLARE2 (Addgene 158700) and tetracycline response element driven expression of either mCherry or eGFP fluorescent reporters (TRE-mCherry (Addgene 92202) or TRE-eGFP (Addgene 89875)) were gifts from M. Sanchez and A. Ting, and were injected using a 10 μl microsyringe with a beveled 33-gauge microinjection needle (Nanofil; World Precision Instruments (WPI)). A total volume of 1.5 µl of virus was injected (750 nl at −1.35 DV and 750 nl at −1.55 DV) at a rate of 100 nl min^−1^ using a microsyringe pump (UMP3; WPI) and its controller (Micro4; WPI). After each injection, the needle was raised 100 μm for an additional 10 min to allow for viral diffusion at the injection site and then slowly withdrawn.

For two-photon and closed-loop optogenetic experiments, the FC was targeted using the following coordinates from the bregma: −1.9 mm AP, +0.15 mm ML and −1.85 mm DV. For two-photon experiments, 60 nl of AAV5-syn-FLEX-jGCaMP8f-WPRE (a gift from the GENIE Project, Addgene 162379) was injected. For closed-loop optogenetic experiments, 60 nl of either AAV1-hSyn1-SIO-stGtACR2-FusionRed (a gift from O. Yizhar, Addgene 105677) or AAV5-EF1a-DIO-mCherry (University of North Carolina Vector Core) was injected. After each injection, the needle was slowly withdrawn.

Mice were selected for viral injections and experimentation with no particular order to avoid systematic biases. Expression was verified after each experiment, and only mice with clear expression were used for further analyses.

### scFLARE labeling in mice

Light was delivered 6–7 days following viral injection. For light delivery, the optical fiber implant was connected to a 473 nm diode-pumped solid-state laser (Shanghai Laser & Optics Century). Mice were allowed 2 h to recover from implant surgery before light delivery. For kainate experiments, closed-loop seizure detection and light delivery were carried out as previously described^22^. Briefly, LFP recording electrodes (PlasticsOne) for kainate-injected animals were connected to an electrical commutator (PlasticsOne) routed to an amplifier (BrownLee 410, Automate Scientific), and in turn connected to a digitizer (USB-6221, National Instruments) and a computer running custom MATLAB recorder and seizure detection software. When a seizure was detected, the software enabled light delivery. Animals in all groups receiving light had one single session of 10 mW 473 nm light delivered in 2 s pulses every 6 s (33% duty cycle), for a total of 10 min. Animals were euthanized and perfused 18–24 h after the end of light administration.

### In vivo two-photon calcium imaging

Within 1 week of virus injection, mice were anesthetized with isoflurane and secured into a stereotaxic frame. We then inserted a 4-mm-long 0.5-mm-diameter gradient-index (GRIN) relay lens (Inscopix), which was lowered with a stereotaxic arm at a 5° angle (to avoid the superior sagittal sinus) to a target of −1.9 mm AP, +0.15 mm ML and −1.7 mm DV, which is at the bottom of the corpus callosum and just above the FC. After at least a week of recovery, a small craniotomy was performed over the ipsilateral hemisphere (−2 mm posterior, −2 mm lateral to the bregma—marked with a permanent marker during the previous surgery) under isoflurane anesthesia and mice were transferred to a floating ball where they woke up. A bipolar twisted wire (A-M Systems, catalog number 795500) tungsten electrode with gold amphenol pin connectors was slowly lowered into the CA1 hippocampal subfield while LFP was monitored for the occurrence of ictal and interictal spiking activity. Once the electrode was at an ideal depth (maximum spiking amplitude), it was secured in place with dental cement and the mouse was returned to its home cage.

Mice were habituated to the imaging setup (a treadmill consisting of a 2-m-long belt) and head fixation for at least two 20 min sessions before experimentation. Ipsilateral CA1 LFP during imaging was amplified 1,000× (A-M systems 1700) and digitized at 10 kHz. Imaging was performed on a two-photon microscope (Neurolabware) equipped with a pulsed infrared laser (Mai Tai, Spectra-Physics) tuned to 920 nm, GaAsP PMT detectors (Hamamatsu) and a ×16 water immersion objective (0.8 NA, 3.0 mm WD; WI, Nikon) and recorded at 15.49 frames per second (Scanbox.org).

Calcium imaging data were processed and analyzed using Python scripts. Motion correction was performed using the HiddenMarkov2D function of SIMA^23^. Binary regions of interest were drawn manually around jGCaMP8f-expressing cells visible on an average intensity projection image of motion-corrected movies. Next, the fluorescence intensity traces were extracted for each region of interest by averaging the included pixel intensities within each frame. Changes in fluorescence intensity (DF/F) traces were obtained as described previously^24^. LFP traces were automatically processed to detect ictal and interictal spiking as described^24^.

### Closed-loop seizure detection and light delivery

Within 1–2 weeks of virus injection, mice were anesthetized with isoflurane and secured into a stereotaxic frame. We then performed a small craniotomy and inserted optical fibers (0.37 NA, low OH, 200 μm diameter; ThorLabs) terminated in 1.25 mm ceramic ferrules (Kientec Systems) to a target of −1.9 mm AP, +0.15 mm ML and −1.7 mm DV, which is at the inferior aspect of the corpus callosum and just superior to the FC. During the same surgery, another small craniotomy was performed over the ipsilateral hemisphere (−2 mm AP, +2 mm ML) and a bipolar depth electrode (PlasticsOne) was implanted to a depth of −2 mm DV to detect seizures from the CA1.

Following the implant procedure, animals were connected through an electrical commutator (PlasticsOne) to a Brownlee 410 amplifier; signals were digitized by an NI USB-6221-BNC digitizer (National Instruments) sampled at 500 Hz and analyzed in real time using a PC running a custom MATLAB seizure detection algorithm as previously described. Animals were also connected to a fiber-coupled diode laser (Shanghai Laser & Optics Century) with 473 nm wavelength to activate the GTaCR2 opsin. Optical patch cords (Thorlabs, Doric Lenses) directed the laser light to the mouse through an optical commutator (Doric Lenses), and were terminated in a 1.25 mm ferrule, which was connected to the implanted optical fiber with a ceramic split sleeve (Precision Fibre Products). Laser power at the source was 0.5 mW.

Continuous LFP monitoring established the presence of spontaneous recurrent seizures in individual animals, at which time an experimenter used custom MATLAB software to identify features of the early ictal electrographic signal to be used in triggering the real-time closed-loop seizure detection software. For semiautomatic analysis of spike clusters (that is, seizures) as previously described^21,22^, the custom MATLAB program used different detection criteria provided by the experimenter for LFP spikes (including filtering, amplitude threshold, width and template matching), LFP spike clusters (including interspike interval and intercluster interval) and artifact rejection (including different filters and signal features), which were combined using Boolean logic. The experimenter verified and, if necessary, corrected all processed files on their detection accuracy of seizure starts and ends. Spike clusters with an interspike interval of less than 1 s were included as seizures. Seizure duration values used for analysis were taken from time of trigger for the closed-loop detector to the end of the seizure. Seizures were not considered ended until the spiking rate fell below one spike every 2 s. The experimenter was blinded to whether a mouse was in the experimental or control group when selecting the inclusion and exclusion criteria and adjusting thresholds to optimally detect seizures.

### Mouse perfusion, histology and imaging

After all data were collected for each mouse, the animals were euthanized by being deeply anesthetized with a mixture of ketamine and xylazine (80–100 mg kg^−1^ ketamine, 5–10 mg kg^−1^ xylazine; intraperitoneal) and transcardially perfused with 10 ml of 0.9% sodium chloride solution followed by 10 ml of cold 4% paraformaldehyde dissolved in phosphate buffer solution. The excised brains were held in a 4% paraformaldehyde solution for at least 24 h before being sectioned into 60 μm slices using a vibratome (Leica VT1200S, Leica Biosystems). For immunostaining, the slices were incubated in blocking buffer containing 1% bovine serum albumin and 0.5% Triton-X in tris-buffered saline (TBS) for 1 h at room temperature, then incubated with rabbit anti-PCP4 antibody (1:200; Sigma HPA005792) in TBS containing 1% bovine serum albumin and 0.5% Triton-X overnight at 4 °C. The slices were subsequently washed in TBS (4 × 10 min) before being incubated in anti-rabbit secondary antibodies conjugated to Alexa Fluor 647 (1:1,000; Thermo Fisher Scientific A-21245) for 2 h at room temperature. Afterward, the slices were washed in TBS (4 × 10 min) before being mounted on glass slides and covered with a coverslip using Vectashield Antifade Mounting Medium (Vector Laboratories). Imaging was performed on a Zeiss LSM 800 confocal microscope using a ×10 or ×20 objective, and a z-stack of 5–7 images was taken.

### Patient selection

Patients 1–4 were considered clinically to have TLE of uncertain laterality and precise anatomical origin, and patient 5 was believed to have an occipital focal cortical dysplasia with subsequent involvement of her mesial temporal lobe. Per routine clinical protocols in our institution, these patients underwent bilateral sEEG recordings after giving informed patient consent. Access to the resulting data followed research procedures approved by the Stanford institutional review board (IRB 70482). Of note, many of the patients also belonged to a cohort that underwent sampling of thalamic targets, as detailed in our previous study^25^. Patient 6 underwent placement of sEEG electrodes as part of routine clinical care owing to seizure recurrence after LITT amygdalohippocampectomy; thus, an electrode was placed in his residual posterior hippocampus as part of normal seizure evaluation. Before the sEEG recordings, all patients completed a comprehensive set of evaluations, including detailed clinical history, neurological examination, neuropsychological assessment, structural magnetic resonance imaging (MRI) and scalp EEG monitoring. Patients completed additional imaging and neurophysiological studies as needed for presurgical planning, including functional MRI for language mapping, fluorodeoxyglucose positron emission tomography (PET) study and high-density electrical source imaging. The sex and gender of human research participants were based on self-reporting. Sex and gender were not considered in the study design. Due to the low number of participants, gender-based analysis was not performed.

### Electrode trajectory planning

The approximate locations and number of electrodes, along with their trajectories, were planned in a multidisciplinary surgical epilepsy conference with a detailed review of presurgical data leading to the clinical hypotheses of most likely seizure onset zones. High-resolution T1, and T1 post-contrast imaging, were used for planning. To sample the posterior hippocampal tail containing the FC, the usual posterior hippocampal target was adjusted further posterior–medially, with the electrode entry point in the inferior temporal gyrus and optimized for a safe, subventricular trajectory. We used only reduced-diameter (0.86 mm) electrodes (Ad-Tech Medical) to help ensure minimal disruption to tissue.

### Intraoperative workflow

Patients 1–6 were brought to the operating room where general endotracheal anesthesia was induced. Five bone fiducials were placed. A volumetric intraoperative O-arm (Medtronic) computed tomography (CT) scan was obtained with the fiducials. The image data set was then merged with the preoperative CT and T_1_ pre- and post-contrast MRI scans. The patient was placed in a Leksell head holder and positioned supine. The ROSA robot (Zimmer Biomet) was then attached to the Leksell adapter and registered to the patient’s head using the bone fiducials. Registration was accepted once <0.5 mm accuracy was achieved. The head was then prepped in the usual fashion. For each percutaneous trajectory, the ROSA robot was positioned coaxially. A small vertical stab incision was made with a number 15 blade. A 2.4 mm drill bit was then introduced through the ROSA drill guide, and the drill guide lowered coaxially all the way down to the scalp. Once through the inner table of the skull, a bolt (bone anchor) was placed. A reduced-diameter (0.8 mm) obturating stylet was passed slowly to create the trajectory. Once the stylet was passed to depth and then removed, a reduced-diameter (0.86 mm) electrode was passed to target depth, the inner stylet was removed and the electrode was tightened into the bolt cap.

### Co-localization of electrodes

A thin-cut CT scan of the head was obtained after electrode implantation to confirm the absence of intracranial hemorrhage. In addition, the CT images were co-registered to MRI data for verification of the trajectory. The electrode coordinates in the native anatomical space were carefully inspected for every single electrode contact and manually labeled by a neurologist and anatomist based on the individual brain’s morphology and landmarks.

### Intracranial recording and identification of ictal patterns

Signals were collected from multiple-contact depth electrodes with a center-to-center contact spacing of 3 mm. A continuous EEG signal was acquired with a digital Nihon Kohden EEG machine at a sampling rate of 1,000 Hz, in combination with continuous video recording. High-frequency filter, time constant and voltage sensitivity settings were adjusted to optimize visual detection of high-frequency oscillations (typically at 300 Hz high-frequency filter, 0.001 s time constant, 10 µV sensitivity). A sEEG bipolar montage including all channels was used for signal detection. Channels with excessive artifacts obscuring EEG signals were excluded from the analysis. All seizures captured were reviewed for onset zones, which were determined by visual analysis by the primary inpatient epilepsy team. Ictal onset signals identified by the epileptologists were inclusive of various morphologies, such as pathologic high-frequency oscillations, evolving fast activity, rhythmic spikes or rhythmic spike–waves. To examine the relationship between seizure activity in the FC and that in the anterior hippocampus, an epileptologist read all files (blinded to whether each LFP trace was from the FC or another region), and when clear ictal patterns were separated by 50 ms or more, a differential onset was described.

### Fiber-optic insertion for LITT

Patient 6 underwent endotracheal general anesthesia in the MRI suite and was placed in a supine position with a bump and head rotation, with his left ear superior. His head was secured to the table of the interventional MR scanner by four skull pins, and the exposed temporal region was clipped, prepped and draped, with the top of his pinna bent inferiorly to expose a low temporal entry site, where a sterile self-adhesive fiducial grid (Clearpoint Neuro) was placed.

Targeting trajectories were planned using a Clearpoint Neuro workstation, and the grid was punctured with a trocar to mark the entry site. The grid was removed, a scalpel was used to make a stab incision and the Clearpoint frame base was affixed to the skull via self-tapping screws. A series of planning scans were obtained to align the frame along the correct trajectory. An MRI-compatible hand drill was then used to make a burr hole through the stab incision, and a ceramic rod was partially inserted to confirm the correct trajectory. Next, a Visualase cooling cannula (with stiffening stylet) was inserted through a reducing cannula, its placement confirmed with imaging. The stylet was removed and the optical fiber was inserted. The laser fiber and cooling lines were connected to the Visualase workstation, and temperature safety limits were set relative to the thermometric monitoring image, in the inferior lateral thalamus (particularly the lateral geniculate nucleus), basal ganglia and lateral mesencephalon, to automatically terminate laser delivery if these structures exceeded 45 °C. The initial lesion was made in the hippocampal tail remnant during real-time MRI thermometry. The laser fiber was then retracted in approximately 1 cm increments, and several overlapping focal ablations formed a tubular ablation zone encompassing the hippocampal tail remnant and FC.

### Data analysis, software and code

Software used for data acquisition included Zen Blue (Zeiss LSM 800 confocal microscope image acquisition), Matlab R2019b (LFP recording, 2p imaging) and Nihon Kohden Neuroworkbench Version 08–11 (patient EEG). Data analysis was performed using the following software: Matlab R2019b, Pycharm Community Edition 2018.2.5, ImageJ 1.53, Graphpad Prism 9, OriginPro 2021b, Python 3.9.7, Pandas 1.3.4, Scipy 1.7.1, Statsmodels 0.13.2, Pingouin 0.5.2 and Seaborn 0.12.1.

### Reporting summary

Further information on research design is available in the Nature Portfolio Reporting Summary linked to this article.

## Online content

Any methods, additional references, Nature Portfolio reporting summaries, source data, extended data, supplementary information, acknowledgements, peer review information; details of author contributions and competing interests; and statements of data and code availability are available at 10.1038/s41591-024-02924-9.

### Supplementary information

Reporting Summary

## Data Availability

Preclinical datasets have been deposited in the Zenodo database: 10.5281/zenodo.10617130 (ref. ^26^).

## Code availability

The analysis code for preclinical data has been deposited in the Zenodo database: 10.5281/zenodo.10617130 (ref. ^26^).
