# Supplementary material for: The fasciola cinereum of the hippocampal tail as an interventional target in epilepsy
Source: Nat Med. 2024 Apr 17;30(5):1292–9. doi: 10.1038/s41591-024-02924-9 (PMC11108783; doi:10.1038/s41591-024-02924-9)
Supplement: Supplementary file 1 — Reporting Summary [file 41591_2024_2924_MOESM1_ESM.pdf]

nature portfolio

Corresponding author(s): Ryan M. Jamiolkowski, Quynh-Anh Nguyen

Last updated by author(s): Feb 28, 2024

Reporting Summary

Nature Portfolio wishes to improve the reproducibility of the work that we publish. This form provides structure for consistency and transparency in reporting. For further information on Nature Portfolio policies, see our Editorial Policies and the Editorial Policy Checklist.

Statistics

For all statistical analyses, confirm that the following items are present in the figure legend, table legend, main text, or Methods section.

n/a	Confirmed	
☐	☒	The exact sample size (n) for each experimental group/condition, given as a discrete number and unit of measurement
☐	☒	A statement on whether measurements were taken from distinct samples or whether the same sample was measured repeatedly
☐	☒	The statistical test(s) used AND whether they are one- or two-sided<br>*Only common tests should be described solely by name; describe more complex techniques in the Methods section.*
☐	☒	A description of all covariates tested
☐	☒	A description of any assumptions or corrections, such as tests of normality and adjustment for multiple comparisons
☐	☒	A full description of the statistical parameters including central tendency (e.g. means) or other basic estimates (e.g. regression coefficient) AND variation (e.g. standard deviation) or associated estimates of uncertainty (e.g. confidence intervals)
☐	☒	For null hypothesis testing, the test statistic (e.g. F, t, r) with confidence intervals, effect sizes, degrees of freedom and P value noted<br>*Give P values as exact values whenever suitable.*
☒	☐	For Bayesian analysis, information on the choice of priors and Markov chain Monte Carlo settings
☒	☐	For hierarchical and complex designs, identification of the appropriate level for tests and full reporting of outcomes
☒	☐	Estimates of effect sizes (e.g. Cohen's d, Pearson's r), indicating how they were calculated

Our web collection on statistics for biologists contains articles on many of the points above.

Software and code

Policy information about availability of computer code

Data collection	Zen Blue (Zeiss LSM 800 confocal microscope image acquisition); Matlab R2019b (LFP recording, 2p imaging); Nihon Kohden EEG machine running Neuroworkbench Version 08-11 (patient EEG); Clearpoint Neuro workstation; Visualase workstation; ROSA robot
Data analysis	Matlab R2019b, Pycharm Community Edition 2018.2.5, ImageJ 1.53, Graphpad Prism 9, Python 3.9.7, Pandas 1.3.4, Scipy 1.7.1, Statsmodels 0.13.2, Pingouin 0.5.2, Seaborn 0.12.1. Data and code used can be found at https://doi.org/10.5281/zenodo.10617130.

For manuscripts utilizing custom algorithms or software that are central to the research but not yet described in published literature, software must be made available to editors and reviewers. We strongly encourage code deposition in a community repository (e.g. GitHub). See the Nature Portfolio guidelines for submitting code & software for further information.

Data

Policy information about availability of data

All manuscripts must include a data availability statement. This statement should provide the following information, where applicable:

- Accession codes, unique identifiers, or web links for publicly available datasets
- A description of any restrictions on data availability
- For clinical datasets or third party data, please ensure that the statement adheres to our policy

Raw preclinical datasets and additional custom code are openly available at https://doi.org/10.5281/zenodo.10617130.

Research involving human participants, their data, or biological material

Policy information about studies with human participants or human data. See also policy information about sex, gender (identity/presentation), and sexual orientation and race, ethnicity and racism.

Reporting on sex and gender	Sex and gender of human research participants was based on self-reporting. Information on patient sex and gender has been provided in Table 1. In total, 4/6 patients were male and 2/6 were female. Sex and gender was not considered in study design. Due to the low number of participants, gender-based analysis was not performed.
Reporting on race, ethnicity, or other socially relevant groupings	Race, ethnicity, or other socially relevant groupings are not reported.
Population characteristics	Population characteristics are described in Table 1 which includes information regarding Age, Sex, Seizure Frequency, Semiology, Current anti-epileptic medications, Prior anti-epileptic medications, MRI diagnosis, PET diagnosis, sEEG findings, and Intervention administered.
Recruitment	Patient 1-4 were considered clinically to have TLE of uncertain laterality and precise anatomical origin, while patient 5 was believed to have an occipital focal cortical dysplasia with subsequent involvement of her mesial temporal lobe. These were consecutive patients over 4 months undergoing sEEG who 1) met that clinical criteria and 2) had a safe trajectory for targeting of the FC based on vascular and ventricular anatomy. All of these patients underwent passive recording with no intervention, and thus no randomization was used. Recordings from different brain regions of the same patient were used as internal controls, and the EEG was interpreted with the reader blinded to which channels were recorded from which contacts.<br><br>Potential bias: Patients with clear unilateral mesial temporal lobe epilepsy with concordant scalp EEG and MRI findings suggestive of hippocampus sclerosis were unlikely to be considered for sEEG, as these patients often times would be "skip candidates" directly to laser ablation. Thus clear unilateral mesial temporal epilepsy would not have been included in this initial cohort. Thus, patients 1-5 demonstrate the FC's role as a seizure focus in a population of less straightforward, more difficult-to-treat TLE patients than those with clear unilateral mesial TLE. Results from these patients should be interpreted with care when applied to patients with straightforward, unilateral mesial TLE.<br><br>In contrast, patient 6 was initially among those more straightforward cases, undergoing his first laser amygdalohippocampectomy prior to any sEEG due to identification as a "skip candidate" from non-invasive studies alone. After seizure recurrence, however, an sEEG electrode was placed in the FC in the remnant hippocampal tail as part of normal clinical care, and the FC was found to be his primary seizure source. Since we only have sEEG recordings from after the initial amygdalohippocampectomy, one should extrapolate that result with care to mTLE patients without a prior intervention.<br><br>Per routine clinical protocols in our institution, and research procedures and consents approved by the Stanford institutional review board (IRB #70482), these patients underwent bilateral stereo-EEG (sEEG) recordings and their data made available for research purposes.
Ethics oversight	Approval for the collection of patient data was provided by the institutional review board at Stanford University (IRB approval number 70482).

Note that full information on the approval of the study protocol must also be provided in the manuscript.

Field-specific reporting

Please select the one below that is the best fit for your research. If you are not sure, read the appropriate sections before making your selection.

☒ Life sciences ☐ Behavioural & social sciences ☐ Ecological, evolutionary & environmental sciences

For a reference copy of the document with all sections, see nature.com/documents/nr-reporting-summary-flat.pdf

Life sciences study design

All studies must disclose on these points even when the disclosure is negative.

Sample size	No statistical methods were used to determine sample size a priori. Sample size was determined based on preliminary experiments that enabled consistent group differences to be identified, and are indicated in figure legends.
Data exclusions	Animals with poor LFP signal or lack of observed seizures during baseline recordings were excluded.
Replication	For all mouse in vivo experiments, replication was performed at least once and showed comparable results. The results from these experiments were pooled and used for analysis.
Randomization	Mice were randomly assigned to each experimental group.
Blinding	LFP recordings were performed without blinding to group allocation because data collection was performed as objectively as possible using the same surgical coordinates, viral titers, and recording conditions. LFP analysis was performed blinded to experimental group.

Reporting for specific materials, systems and methods

We require information from authors about some types of materials, experimental systems and methods used in many studies. Here, indicate whether each material, system or method listed is relevant to your study. If you are not sure if a list item applies to your research, read the appropriate section before selecting a response.

Materials & experimental systems

n/a	Involved in the study
☐	☒ Antibodies
☒	☐ Eukaryotic cell lines
☒	☐ Palaeontology and archaeology
☐	☒ Animals and other organisms
☒	☐ Clinical data
☒	☐ Dual use research of concern
☒	☐ Plants

Methods

n/a	Involved in the study
☒	☐ ChIP-seq
☒	☐ Flow cytometry
☒	☐ MRI-based neuroimaging

Antibodies

Antibodies used

rabbit anti-PCP4 antibody (1:200; Sigma HPA005792) ; anti-rabbit-AlexaFluor647 secondary (1:1000; Thermo Fisher Scientific A-21245)

Validation

-PCP4: Prestige Antibodies® are highly characterized and extensively validated antibodies with the added benefit of all available characterization data for each target being accessible via the Human Protein Atlas portal linked just below the product name at the top of this page. The uniqueness and low cross-reactivity of the Prestige Antibodies® to other proteins are due to a thorough selection of antigen regions, affinity purification, and stringent selection. Prestige antigen controls are available for every corresponding Prestige Antibody and can be found in the linkage section.
Every Prestige Antibody is tested in the following ways:
IHC tissue array of 44 normal human tissues and 20 of the most common cancer type tissues.
Protein array of 364 human recombinant protein fragments.
Corresponding Antigen APREST70287.
Selected references: 1) Blake J Laham et al. Newborn mice form lasting CA2-dependent memories of their mothers. Cell reports, 34(4), 108668-108668. 2) Felix Leroy et al. A circuit from hippocampal CA2 to lateral septum disinhibits social aggression. Nature, 564(7735), 213-218. 3) Saikat Ray et al. Complementary Modular Microcircuits of the Rat Medial Entorhinal Cortex. Frontiers in systems neuroscience, 11, 20-20.

-anti-rabbit-AlexaFluor647: To minimize cross-reactivity, these goat anti-rabbit IgG whole antibodies have been cross-adsorbed against bovine IgG, goat IgG, mouse IgG, rat IgG, and human IgG. Cross-adsorption or pre-adsorption is a purification step to increase specificity of the antibody resulting in higher sensitivity and less background staining. The secondary antibody solution is passed through a column matrix containing immobilized serum proteins from potentially cross-reactive species. Only the nonspecific-binding secondary antibodies are captured in the column, and the highly specific secondaries flow through. The benefits of this extra step are apparent in multiplexing/multicolor-staining experiments (e.g., flow cytometry) where there is potential cross-reactivity with other primary antibodies or in tissue/cell fluorescent staining experiments where there are may be the presence of endogenous immunoglobulins.
Selected references: 1) Xiaowei Sun et al. The endoribonuclease Arlr is required to maintain lipid homeostasis by downregulating lipolytic genes during aging. Nature Communications, 14 (1), 6254. 2) Margarete M Karg et al. Microglia preserve visual function loss in the aging retina by supporting retinal pigment epithelial health. Immunity & Ageing : I & A, 20 (1), 53. 3) Michelle Carmen Jentzsch et al. A New Preclinical Model of Retinitis Pigmentosa Due to Pde6g Deficiency. Ophthalmol Sci, 3 (4), 100332.

Animals and other research organisms

Policy information about studies involving animals; ARRIVE guidelines recommended for reporting animal research, and Sex and Gender in Research

Laboratory animals

C57BL/6 mice (Jackson Labs Strain #664 for Stanford, 12-20 week old) or PCP4-Cre mice (strain #RBRC05662, gift from T. Takemori and A. Ishige [RIKEN, Japan], 12-24 week old).

Wild animals

Study did not involve wild animals.

Reporting on sex

While both male and female mice were used in this study, separating data generated from male and female mice would have made sample sizes too small to draw any meaningful insights or conclusions.

Field-collected samples

Study did not involve samples collected from the field.

Ethics oversight

All experimental protocols were approved by the Administrative Panel on Laboratory Animal Care of Stanford University (Protocol #30183).

Note that full information on the approval of the study protocol must also be provided in the manuscript.

Plants

Seed stocks	N/A
Novel plant genotypes	N/A
Authentication	N/A
